# The lived experience of juvenile idiopathic arthritis in young people receiving etanercept

**DOI:** 10.1186/s12969-016-0083-7

**Published:** 2016-04-12

**Authors:** P Livermore, D Eleftheriou, LR Wedderburn

**Affiliations:** Rheumatology Department, Great Ormond Street Children’s Hospital, Great Ormond Street, London, WC1N 3JH UK; Arthritis Research UK Centre for Adolescent Rheumatology at UCL UCLH and GOSH, and Infection, Inflammation and Rheumatology Section, Institute of Child Health, UCL, London, UK

**Keywords:** Phenomenology, Paediatric, Juvenile idiopathic arthritis, Etanercept, Qualitative, Expectations

## Abstract

**Background:**

This study explores young people’s daily experiences of living with Juvenile Idiopathic Arthritis (JIA) and their thoughts, beliefs and feelings related to the biological drug Etanercept, prescribed as part of their treatment.

**Methods:**

An Interpretive Phenomenological approach was used to allow in-depth examinations of the young people’s personal accounts of their lived experiences. Data were obtained from 6 young people between the ages of 10–13 years, from one tertiary institution’s Paediatric Rheumatology department using audio-taped open-ended interviews.

**Results:**

The transcripts yielded seven thousand words of data and two hundred significant statements, which were reduced to five themes; 1) Who understands me, 2) Medicines and injections, 3) Challenges of schooling and friendships, 4) Being different, and 5) Exclusion from sports. There were marked similarities between the young people’s statements; however, there were also some striking differences. The theme ‘Who understands me’ yielded the biggest section of data, but also produced the biggest disparity between the young people. Two patients were very clear that they thought everyone ‘understands’, whilst two other patients held the belief that ‘no one understood’. This paper explores these statements in further detail.

**Conclusions:**

The findings from this study can give healthcare professionals novel insight into the likely reactions to-treatment for JIA and, through this, enable them to offer improved support, education and early intervention before these issues become a concern. This study also provides insight into the emotional resilience of young people with JIA.

**Electronic supplementary material:**

The online version of this article (doi:10.1186/s12969-016-0083-7) contains supplementary material, which is available to authorized users.

## Background

Juvenile Idiopathic Arthritis (JIA) is one of the most common autoimmune diseases of childhood, and is one of the leading causes of childhood-acquired disability. It is increasingly recognised that JIA has major health consequences that impact on social, educational and family life throughout the teenage years and well into adulthood [1, 2]. Over the past decade the advent of new therapies for paediatric rheumatic conditions, particularly the introduction of biological agents, has radically changed the management of patients with JIA. Etanercept, a tumour necrosis factor (TNF)-alpha receptor antagonist (anti-TNF), was the first biological agent approved for use in JIA, and is given either as a twice or once weekly subcutaneous injection [3]. According to the UK National Institute of Clinical Excellence (NICE) guidelines published in 2002 only young people who have failed Methotrexate treatment (either due to lack of efficacy or intolerability) may be prescribed Etanercept [4]. However, this medication provides no guarantee of success; initial studies suggest approximately 70-75 % of young people improve on biological therapies and the response rate is lower than this in some subtypes of JIA [5, 6].

Although the biological efficacy of Etanercept is established, how children and young people feel when receiving the therapy is unexplored. Some research has been conducted to explore the psychological responses to success or failure of drug treatment in adult patients, but few studies so far have focussed on young people receiving biologic therapies and how they assess its impact. Hence, the aim of this study was to understand the young people’s perspectives of living with JIA specifically receiving therapy with biologic agents.

## Methods

Interpretive Phenomenology was the chosen method for this research, as the goal of phenomenology is to study how people make meaning of their lived experience and to answer the question ‘what does it feel like?’ [7] The methodology chosen supports the desire to gain greater insight to the lives of patients and the impact their illness has upon them, inviting them to offer descriptions of their experiences and discuss their views of the medication they are receiving. Phenomenological studies typically involve small numbers of participants, often no more than ten.

### Patient population

This study was a sub-study of the Childhood Arthritis Response to Medication (CHARMS) Study [8, 9]. Full ethical approval was sought and full informed parental consent and young person assent were obtained for all the subjects. The CHARMS study collects data in two arms, a prospective and retrospective group, studying biological mechanisms of, and psychological responses to, success or failure of drug treatment in JIA. As part of this wider sponsored study, this project provided a mechanism whereby further in-depth qualitative data could be collected from a subset of subjects.

### Data collection

**Fig. 1:**
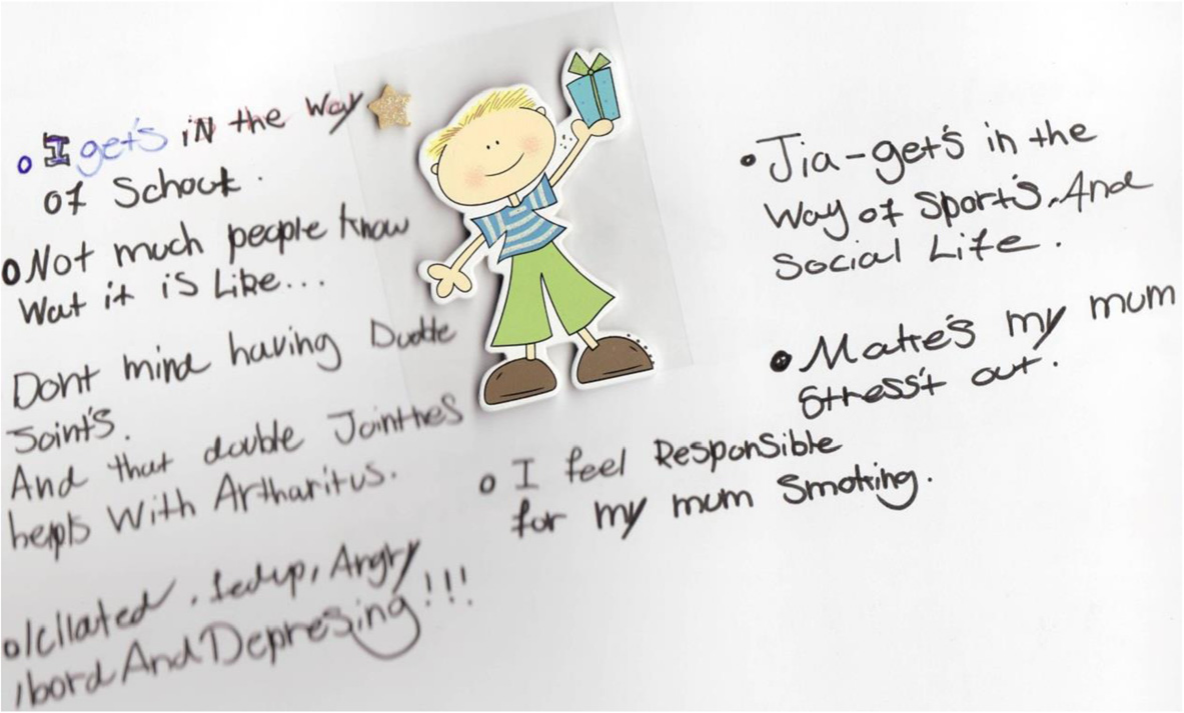
A spider diagram drawn by one of the young participants. This is one of the spider diagrams written by one of the young people. The young person used this space to write about how their arthritis makes them feel. The sticker in the middle was for the young person to visualise themselves in the middle, and the words written around the outside describe how they feel and their concerns. Some examples of the text written here include such comments as; ‘Isolated, fed up, angry, bored and depressing’, ‘I feel responsible for my mum smoking’ and ‘JIA gets in the way of sports and social life’

Data were obtained from young people attending a tertiary care Paediatric Rheumatology Department, using audio-taped open-ended semi-structured interviews aided by spider diagrams drawn by the young person. Purposive sampling captured young people who were receiving their first anti-TNFmedication. The six participants who were approached met the inclusion criteria of a) having a diagnosis of JIA, b) on Etanercept, c) ten years of age or over. There were no refusals or withdrawals. All young people chose to be interviewed on their own. Each interview had the same structure, and began by asking the young person how they felt about their arthritis, and then asking them to either draw/write (Fig. 1) or discuss this in more detail. Each interview naturally progressed as young people talked about what was important to them. Each interview was between 30–60 min long.

### Analysis

Analysis of the data was undertaken using Colaizzi’s seven stage process; this method was chosen due to its compatibility with the interpretive approach [10, 11]. The first stage of Colaizzi’s (1978) process is to transcribe the tapes word for word and then to read and re-read the typed narratives. Each transcript is analysed to identify significant statements that told each participant’s story of their lived experience [12]. At this stage it is important to highlight as many statements as possible within each transcript and to be observant for the manner in which each statement was iterated, “empathetically dwelling” with each experience [11]. Formulated meanings were devised for each participant and arranged on a separate sheet; this ensured the young person’s own words constituted the formulated meaning. Throughout the whole process, constant reference was made back to the young person’s spoken words to maintain credibility. Eventually five themes were identified with all young people having a voice in each theme.

When the seventh stage of data analysis was approached (return to the subject and check they agree with the findings) the lead researcher was concerned about ethical implications of returning the text to the young people, for concern of stirring up emotions. It was therefore decided to produce a newsletter, to encompass an overview of all the young people’s comments, but also to be a resource of further advice and help, such as websites. This way the young people, who had been going through a particularly difficult time, should not be reminded of how they felt, but in keeping with Colaizzis (1978) view that they the young people are co-researchers and should be involved, they would still have some feedback from their participation.

## Results

### Interview data

The 6 participants recruited were aged 10–13 years, 2 female, 4 male, all Caucasian, had English as first language, and disease duration of 5–11 years. All patients were receiving both Methotrexate and Etanercept, and this was their first anti-TNF therapy. Two young people had polyarticular JIA, 2 had systemic JIA and 2 had extended oligoarticular JIA by internationally agreed classification criteria [13] (Demographics are shown in Additional file 1: Table S1). In relation to the decision to include two young people with Systemic JIA, it is worth noting that these children now had polyarticular disease with no recent systemic features. This study aimed to be inclusive, capturing young people’s views on their arthritis and biological medication, so the view of these individuals was felt to be just as valid as others, although we acknowledge that prior experience in those with sytemic JIA may be different from those with Polyarticular JIA at onset. All patients had started Etanercept within a year (range 2–10 months) of this study. At the time of interview, the number of current active joints ranged from 1–9 in all the young people, none were in remission. One of the young people had previously had a hip replacement, but there were no other co-morbidities, such as uveitis or pain syndromes. As this was a small indepth study looking at young peoples thoughts, Health Related Quality of Life data was not collected, however all young people had significant disease of a minimum of five years of disease duration and the need to attend at least 3 monthly clinics. The comments, thoughts, perceptions and feelings of the young people fell into 5 themes, which for this analysis were called: who understands me?; medicines and injections; challenges to schooling and friendships; being different; and exclusion from sports, especially physical education (PE). These data are each presented here in turn.

### Themes

#### Who understands me?

This section of data yielded the largest cluster and showed the biggest disparity between the young people. Two of the young people voiced the opinion that others don’t understand what it is like to have arthritis, whereas the other four talked about some people understanding, such as close family. For example, Patient 1 said “in school they didn’t really understand, and then my mum got this video and then everyone started understanding and stopped asking questions”. Patient 2 said “my five year old sister understands what I’ve got wrong with me”. In this section Patient 3 said “I feel angry, angry all the time, angry that no-one understands me, it’s very lonely to have arthritis”; patient 4 said “it’s sad and depressing’cuz people just stay away from you, they don’t know that people and young kids can get it too, they don’t believe you and think you are making it up because they only know about old grannies”. Patient 5 said “no one knows what I’ve been through”.

#### Medicines and injections

This theme revealed marked similarity in the young people’s comments. Five of them remarked on positive differences between Etanercept and their previous therapy, and whilst injections were highlighted as a drawback by all of the young people, some coped better than others. Patient 1 commented that “the Etanercept doesn’t sting as much and the needles are smaller”, Patient 6 said that “I have to inject it into myself, it doesn’t bother me, I used to inject my Methotrexate and Anakinra, so it’s all the same”. Patient 2 seemed confused about side effects by saying that “this drug’s ok, the methotrexate never worked’cuz I was always sick (made me vomit)” and, Patient 5′s observation was that “I don’t want to give it to myself”. Finally, Patient 3′s statement was that “the Etanercept’s useless’cuz it doesn’t work for me, I’m just a guinea pig, you get told all these things will work, they don’t, nothing works”.

### Challenges to schooling and friendships

None of the patients could separate schooling and peer relationships from their arthritis; all faced challenges with their education and/or friendships. Some comments were specific to actual school facilities and the environment. Patient 1 said “I have a laptop because I can’t write quickly” and Patient 4 said “it’s hard to get around the school when you are on crutches as the corridors are too thin”. Patient 6 and Patient 3 talked more about friendships at school, Patient 6 “my mates are really good, they know what is wrong with me” whereas Patient 3 said “I feel lonely, it affects your friendships, secondary school is so different, they even have a disability office, loads of wheelchairs and special glass lifts–makes you feel sad”. Patient 5 mentioned the effects of being absent: “I didn’t go to school for about 6 months, I didn’t do any work, I get low school reports because I’m not there”.

### Being different

This theme yielded much of the strong emotion that was expressed, as illustrated from each of the following significant statements. Patient 1: “it’s annoying to see other children running about and you think I wish I was there”. Patient 6: “I wish I didn’t have it, I always wonder what I would be like, how my family would treat me differently”. Patient 3: “it’s always there in your mind that you are not normal”. Patient 2: “you have to do exercises every night which hurt”. Patient 4: “people treat you differently, the idea of being different is upsetting, well not that different, being different is quite a big thing, I don’t want extra help as I don’t want to be more different, people automatically think you can’t do things and you can’t be bothered to say you can, so you end up sitting watching and feeling sad”. Patient 5: “you don’t feel confident to socialise with your friends, you just feel different, like you don’t really fit in, makes you feel isolated and fed up”.

### Exclusion from sports, especially Physical Education (PE) lessons

This theme, whilst being the smallest cluster of significant statements, had a premise universal for all six young people–that of not being able to fully participate in sports, especially physical education (PE). Patient 4 commented that “I get out of PE as I can’t do it at times, and people think I just use it as an excuse, I now go to the library and stamp books instead”. Patient 6 said “I can’t do rugby and football now I’ve had both my hips replaced” and Patient 3 reflected “no point even talking about football anymore, I can’t wear my kit as I can’t play, I can’t even do it now, I wouldn’t be good, not as good as if I didn’t have this–the arthritis, ‘my devil’, no point bringing friends back to the house, we used to play football in the garden, it’s what we used to do, but until I’m normal again, I don’t want to keep making excuses”.

## Discussion

In this study, an in-depth open but semi-structured interview approach has facilitated the uncovering of many mixed emotions of young people with arthritis. ‘Who understands me’ was a particularly complex theme, as the term ‘understanding’ covers many aspects of a chronic disease. One of the points raised in this section was the view that ‘no one knows kids can get arthritis too because they only know about old grannies’. This created frustration in trying to explain to friends and teachers about their illness. This is an issue raised by Hutchinson and Hall 2007 [14].

In the theme of ‘Medicines and Injections’, most of the young people were positive about their Etanercept therapy. This supports the study by Marshall et al. [15], in which the majority of adult patients were very positive about the effects of their treatment. Whilst they found positive statements were related to patients’ perceived improvement in quality of life, in our present study young people were more positive about the difference in side effects between Etanercept and their previous therapy, Methotrexate. This issue is not discussed by Marshall et al. 2004 [15]. One issue that arose for one patient was being ‘promised’ that the drug would work, and the patient feeling it hadn’t. This supports Marshall et al. 2004 [15], who state that some patients in their study found that anti-TNF therapy did not live up to their high expectations.

‘Challenges to schooling and friendships’ covered many different issues. All of the young people mentioned missing school to go to hospital. The advice booklet “When a Young Person has Arthritis: for teachers” [16] specifically highlights provision and support a young person may need at school; however, it does not mention the young person’s fear of asking for these provisions. Whilst some young people may be happy to have a laptop when they found handwriting was difficult, some of the others in the study were quite concerned about extra assistance making them appear different. The issue of transition from primary to secondary school was mentioned by three of the young people, who all alluded to the negatives of moving from primary to secondary, and were anxious about this change. Three of the young people indicated feelings of ‘loneliness’; one of the tasks of adolescence is ‘making friendships’ and finding out where you fit with your peer group and it was these young people recently moved to secondary school who were especially struggling with their friends.

In the theme defined as ‘Being different’ all of the young people acknowledged that they felt ‘different’ from peers and highlighted that they did not wish to feel this. Whilst ‘feeling different’ does not have to be a negative emotion, in the present study it was felt that these six young people all saw it as such: ‘feeling different and then sad’ or ‘feel different from how a normal person feels’. These comments could be a concern to health care professionals and lead them to consider whether these young people need extra support [17].

When discussing sports and PE lessons, the message was the same from all the subjects: we do not participate in sport as our friends do. Paradoxically, there are many reasons why young people with JIA need to do daily exercises, including to help maintain a better range of joint movement and mobility, to prevent contractures, to prevent osteoporosis and to build up muscle strength [18], yet often they do not take part in school PE.

### General applicability

There are three particular areas which it is felt that we as health care professionals could improve upon: firstly, transition between primary and secondary (or ‘high’) school. Here, simple measures may help, such as discussing this transition in advance when young people are still at primary school, reviewing the role of the school nurse in terms of support, and highlighting for families what a difficult time this can be. Exclusion from sports, especially PE, was the second issue highlighted by all of the children, not just the males, and it is the responsibility of the clinician to identify those not fully partaking in PE at routine appointments, to highlight the benefits of joining in PE (both physically and psychologically) and to encourage young people to talk about the difficulties they face with sport. The health professional could suggest to the school that modifying the PE class so that the young person with JIA can participate to the best of their ability, may be better than singling them out and giving them another unrelated task to do. Finally, the high expectations of new therapies is the third area identified. Clinicians can help by beginning to discuss changing therapies early on (before the first therapy stops working), to aim to balance high hopes with realistic expectations and to offer young people the opportunity to talk to other young people with similar conditions on similar therapies.

Overall this study has highlighted that young people with JIA can feel isolated and feel that people do not understand them. It is therefore important for the health professional to acknowledge this and offer psychological support early to help young people cope better.

This study has some limitations. “Due to different populations and cultural differences concerning priorities in different countries, this study may have limited applicability outside the United Kingdom.” Qualitative studies are typically small in number, but rich in depth. The study did not have the breadth to be able to further examine differences between the young people’s assessments of the effects of disease duration, adolescent development stage or their individual support networks or collect such data as quality of life and pain scores. The patients seen at our tertiary centre do have severe disease as can be seen in these young people, for example having bilateral hip replacements, and thus their comments may not be typical of a less severe cohort of young people with JIA. Including more males than females and two children with polyarticular systemic disease was purely by chance. We recognise that these 6 cases do not reflect the prevalence of subtypes or gender of these forms of JIA in the population. Given the small sample size we were not able to analyse the effect of gender or JIA subtype within our data; however, it was not felt either of these variables affected the themes expressed or data as a whole in a negative manner. A larger study would be valuable to explore whether gender or JIA subtype may influence the reported experience of biologic therapies in young people with JIA. The fact that the patients were known to the researchers and that all the young people were keen to participate and seemed to enjoy telling their story, gave the study a positive bias.

### Conclusions

This study has highlighted areas where there is a distinct lack of available literature and where further work is needed to fully assist young people with JIA. Future studies of the paediatric rheumatology community are warranted; specifically concerning young people’s lived experiences of JIA and their expectations and experiences of taking a biological therapy. It is important also to raise the profile of JIA in young people, so it is not seen as a disease only of the elderly, possibly by getting young people themselves to be involved in promoting awareness of JIA in young people and thus educating those around them.

Having JIA as a young person can present many challenges. The findings from this study can aid the healthcare professional to have a deeper insight into these and, through this, to be able to offer improved support, education and earlier intervention before these issues become a concern. As highlighted earlier, young people’s perception of their JIA is an area where there is little published research. This study has specifically focused on young people with severe arthritis defined by the need for anti-TNF therapy, therefore this paper is an encapsulation of a very specific cohort of young people; those aged between 10–13 years old, seen in one tertiary institution, with severe arthritis, and receiving a particular therapy. Whilst this limits the generalisability of the findings and requires the researcher to be transparent and make this clear to the reader, it does not detract from the need to ask young people their story and for young people to tell it.

## Additional file

Additional file 1: Table S1. Demographics of all patients captured at time of interview to provide the reader with more clarity of the cohort enrolled on this study. The table shows the data collected such as medications and disease activity at time of interview. (DOC 37 kb)

## References

[1] Ravelli A (2004). Toward an understanding of the long-term outcome of juvenile idiopathic arthritis. Clin Exp Rheumatol.

[2] Foster H, Brogan P (2012). Paediatric Rheumatology.

[3] Woo P, Laxer RM, Sherry DD (2007). Pediatric rheumatology in Clinical Practice.

[4] National Institute for Clinical Excellence (NICE) (2002). Guidance on the use of etanercept for the treatment of juvenile idiopathic arthritis. Technology Appraisal Guidance – No. 35 March 2002.

[5] Lovell DJ, Giannini EH, Reiff A, Cakwell GD, Silverman ED, Nocton JJ, Stein LD, Gedalia A, Ilowite NT, Wallace CA, Whitmore J, Finck BK (2000). Etanercept in children with polyarticular Juvenile Rheumatoid Arthritis. N Engl J Med.

[6] Quartier P, Taupin P, Bourdeaut F, Lemelle I, Pillet P, Bost M, Sibilla J, Kone-Paut I, Gandon-Laloum SG, Lebideau M, Bader-Meunier B, Mouy R, Debre M, Landais P, Prieur AM (2003). Efficacy of etanercept for the treatment of juvenile idiopathic arthritis according to the onset type. Arthritis Rheum.

[7] Starks H, Trinidad SB (2007). Choose your method: A comparison of phenomenology, discourse analysis, and grounded theory. Qual Health Res.

[8] Mulligan K, Etheridge A, Kassoumeri L, Wedderburn LR, Newman S (2009). Do mothers and fathers hold similar views about their child’s arthritis?. Arthritis Rheum.

[9] Moncrieffe H, Ursu S, Holzinger D, Patrick F, Kassoumeri L, Wade A, Roth J, Wedderburn LR (2013). A subgroup of juvenile idiopathic arthritis patients who respond well to methotrexate are identified by the serum biomarker MRP8/14 protein. Rheumatology.

[10] Colaizzi P, Valle R, King M (1978). Psychological research as the phenomenologist views it. Existential Phenomenological Alternatives for Psychology.

[11] Jiang R, Chou C, Tsai P (2006). The Grief Reactions of Nursing Students Related to the Sudden Death of a Classmate. J Nurs Res.

[12] Beck CT (2004). Post-Traumatic Stress Disorder Due to Childbirth. Nurs Res.

[13] Petty RE, Southwood TR, Manners P (2004). International League of Associations for Rheumatology classification of juvenile idiopathic arthritis: second revision, Edmonton, 2001. J Rheumatol.

[14] Hutchinson E, Hall C (2007). A Phenomenological exploration of the patient learning experiences of 16–19 year-old women accessing a young people’s rheumatology service in the UK. J Res Nurs.

[15] Marshall NJ, Wilson G, Lapworth K, Kay LJ (2004). Patients’ perceptions of treatment with anti-TNF therapy for rheumatoid arthritis: a qualitative study. Rheumatology.

[16] ARC (Arthritis and Rheumatism Council). *When a young person has arthritis*. ARC Booklet for teachers; 2003. file:///C:/Users/polly_000/Downloads/2048-When-a-young-person-has-arthritis.pdf

[17] Rosenzweig KJ, Nabors L (2013). Pain coping strategies for children with arthritis. Bio Med Res Int.

[18] Britton C (2003). Kids with Arthritis: A guide for families.

