# Supplementary material for: The lived experience of juvenile idiopathic arthritis in young people receiving etanercept
Source: Pediatr Rheumatol Online J. 2016 Apr 12;14:21. doi: 10.1186/s12969-016-0083-7 (PMC4828872; doi:10.1186/s12969-016-0083-7)
Supplement: Additional file 1: Table S1. — Demographics of all patients captured at time of interview to provide the reader with more clarity of the cohort enrolled on this study. The table shows the data collected such as medications and disease activity at time of interview. (DOC 37 kb) [file 12969_2016_83_MOESM1_ESM.doc]

Additional file 1: Table S1. Demographics at time of interview: (data shown as median (IQR) unless stated otherwise)

| Demographics | Gender | 2 female – 4 male |
| --- | --- | --- |
|  | Age | 12 (10-13) |
|  | Age of onset of disease | 2 (1.5-4.25) |
|  | Disease duration | 8.5 (6.5-10.25) |
|  | JIA type | 2 poly JIA, 2 extended oligo JIA, 2 systemic JIA |
| Medications | Etanercept | 6/6 (Started within the year 2-10 months) |
|  | Prednisolone | 2/6 (both 5mg daily) |
|  | Methotrexate | 6/6 (4 subcutaneous, 2 oral) |
| Disease activity | Remission | 0/6 |
|  | Active joint count | 3.0 (1.75-6.75) |
|  | CHAQ | 1.7 (1.5-2.125) |
|  | Patient VAS (0-10cm) | 3.2 (2.675-6.675) |
|  | Physician VAS (0-10cm) | 2.6 (1.875 – 5.65) |
| Co-morbidities | Major health conditions | 0 |
|  | Uveitis | 0 |
|  | Pain syndromes | 0 |
|  | Other | 1 bilateral hip replacements |
| Social | Living with both parents | 5/6 |
|  | Living with one parent | 1/6 |
|  | School | 6/6 attending school regularly |
| Other Health Care Professionals involved | Psychology | 2 had psychology input (1 of those currently) |
